# Applications of Magnetotactic Bacteria, Magnetosomes and Magnetosome Crystals in Biotechnology and Nanotechnology: Mini-Review

**DOI:** 10.3390/molecules23102438

**Published:** 2018-09-24

**Authors:** Gabriele Vargas, Jefferson Cypriano, Tarcisio Correa, Pedro Leão, Dennis A. Bazylinski, Fernanda Abreu

**Affiliations:** 1 Instituto de Microbiologia Paulo de Góes, Universidade Federal do Rio de Janeiro, Avenida Carlos Chagas Filho, 373, CCS, UFRJ, Rio de Janeiro, RJ 21941-902, Brazil; gabriele@micro.ufrj.br (G.V.); jeff.tm@gmail.com (J.C.); tcorrea@micro.ufrj.br (T.C.); pedroleao@micro.ufrj.br (P.L.); 2 School of Life Sciences, University of Nevada at Las Vegas, Las Vegas, NV 89154-4004, USA; dennis.bazylinski@unlv.edu

**Keywords:** magnetotactic bacteria, magnetosomes, magnetite nanocrystals, biotechnology, nanotechnology

## Abstract

Magnetotactic bacteria (MTB) biomineralize magnetosomes, which are defined as intracellular nanocrystals of the magnetic minerals magnetite (Fe_3_O_4_) or greigite (Fe_3_S_4_) enveloped by a phospholipid bilayer membrane. The synthesis of magnetosomes is controlled by a specific set of genes that encode proteins, some of which are exclusively found in the magnetosome membrane in the cell. Over the past several decades, interest in nanoscale technology (nanotechnology) and biotechnology has increased significantly due to the development and establishment of new commercial, medical and scientific processes and applications that utilize nanomaterials, some of which are biologically derived. One excellent example of a biological nanomaterial that is showing great promise for use in a large number of commercial and medical applications are bacterial magnetite magnetosomes. Unlike chemically-synthesized magnetite nanoparticles, magnetosome magnetite crystals are stable single-magnetic domains and are thus permanently magnetic at ambient temperature, are of high chemical purity, and display a narrow size range and consistent crystal morphology. These physical/chemical features are important in their use in biotechnological and other applications. Applications utilizing magnetite-producing MTB, magnetite magnetosomes and/or magnetosome magnetite crystals include and/or involve bioremediation, cell separation, DNA/antigen recovery or detection, drug delivery, enzyme immobilization, magnetic hyperthermia and contrast enhancement of magnetic resonance imaging. Metric analysis using Scopus and Web of Science databases from 2003 to 2018 showed that applied research involving magnetite from MTB in some form has been focused mainly in biomedical applications, particularly in magnetic hyperthermia and drug delivery.

## 1. Introduction

Magnetotactic bacteria (MTB) represent a diverse group of Gram-negative motile, aquatic microorganisms that have the ability to biomineralize intracellular, nano-sized magnetic crystals, called magnetosomes, through a controlled biomineralization process [1]. These organisms were discovered based on their magnetic response in magnetic fields, called magnetotaxis, where cells passively align and swim along magnetic field lines resulting in their accumulation at the edge of water drops in a magnetic field when viewed with a microscope [2,3]. Although Bellini (1963) postulated that these “magnetosensitive bacteria” possessed an internal “magnetic compass” [3], this was not shown until Blakemore (1975), using transmission electron microscopy, demonstrated the presence of magnetosomes, showing them to be responsible for magnetotaxis, and also coined the term MTB [4]. It is currently believed that magnetotaxis, in conjunction with chemotaxis, aids MTB in locating an optimal position in vertical chemical and redox gradients for survival and reproduction [5]. The discovery of and subsequent research on MTB has been instrumental in answering many important questions regarding the process of biomineralization and the evolution of MTB, as well as the environmental roles these organisms play in natural habitats. Over the years, however, MTB and their magnetosomes have also been the focus of investigations in their use in biotechnological, medical and other possibly commercial applications.

**Figure 1:**
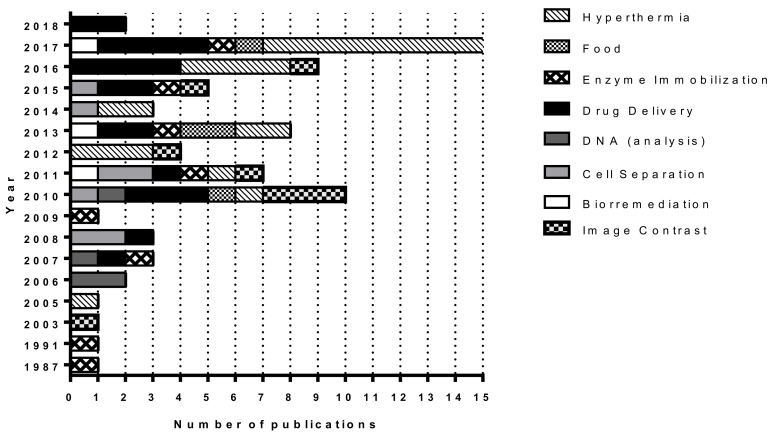
Classification of scientific publications reporting on applications of magnetosomes by area [7,8] from 1987 until 2018. Scientific articles containing the keyword “magnetosome (s)” in the title were classified in different subareas of Biotechnology (bioremediation, cell separation, DNA analysis, drug delivery, enzyme immobilization, food, hyperthermia, image contrast). For analysis of raw data, see Table S1. The criteria used for selection of these data are detailed in “Database generation & analysis section”.

The first MTB cultivated in axenic culture was a magnetite-producing spirillum named *Aquaspirillum* (now *Magnetospirillum*) *magnetotacticum* strain MS-1 [4]. The difficulty in isolating and cultivating new species of MTB hindered research in these organisms for a long period of time, although, over the last 30 years, a number of other, diverse species of MTB have been isolated in axenic culture due to a number of significant improvements in culture methods for new species of MTB particularly since 2011 [6]. In addition, also since 2011, the number of publications focused on the use of MTB and magnetosomes in different biotechnological applications has also significantly increased (Figure 1). Currently there are approximately 25 species of MTB in axenic culture [6]. Despite this, however, few species are available in cell line repositories, including the American Type Culture Collection (ATCC) or the Leibniz Institute DSMZ-German Collection of Microorganisms and Cell Cultures (Deutsche Sammlung von Mikroorganismen und Zellkulturen GmbH; DSMZ).

**Figure 2:**
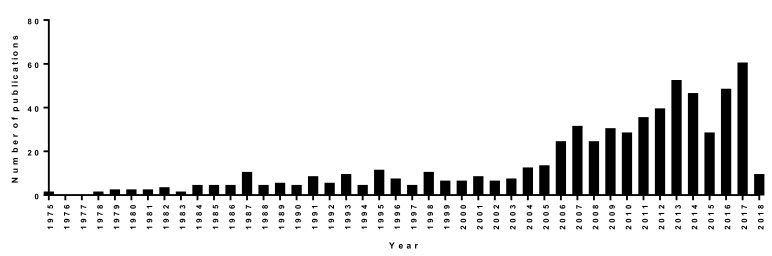
Global publication records (1st January 1975–25th February 2018) containing the keywords “magnetotactic bacteria or magnetosome(s)” in article titles [7,8]. For analysis of the raw data, see Table S2. The criteria used for selection of data are detailed in the “Database generation & analysis section”.

A comprehensive search on Web of Science [7] and Scopus [8] online databases returned a total of 644 articles (original research and reviews) containing the words “magnetotactic bacteria” or “magnetosome(s)” in the title. Publication dates ranged from 1975 (the date of Blakemore′s first publication documenting MTB) until February 2018. Overall, during this period, the number of publications involving MTB and magnetosomes increased significantly (Figure 2). Among these publications, 81 are related to applications of MTB and/or magnetosomes, corresponding to ~12.6% of all scientific papers on MTB since 1975. The majority of these articles deal with applications of MTB and/or magnetosomes in biomedicine, although other applications have also been investigated, for example, in bioremediation [9,10].

The first described application using MTB, in this case magnetosomes, was published in 1987 [11]. In this work, bacterial magnetite magnetosomes, purified from uncultured MTB from a pond (likely a very tedious task!), were used in the immobilization of the enzymes glucose oxidase and uricase. These enzymes showed a 40 times higher activity when immobilized on magnetosomes compared to those immobilized on crystals of chemically-produced magnetite.

A year later, Gorby et al. [12] described a method for the purification of magnetosomes from cells of the cultured MTB, *Ms. magnetotacticum*. Cells were disrupted (lysed) in a French pressure cell press and their magnetosomes purified by magnetic concentration. This procedure was a very important development for several reasons. Not only did it show that MTB could be mass cultured to high cell yield and that studies could now be performed on relatively large amounts of purified magnetite magnetosomes but also that purified magnetosomes could now be feasibly tested in specific applications. Presently, the most common species of MTB utilized in studies involving the mass culture of MTB and the purification of magnetite magnetosomes for applications are *Ms. magneticum* strain AMB-1 and *Ms. gryphiswaldense* strain MRS-1, both of which are relatively easy to grow in mass culture compared to another MTB.

**Figure 3:**
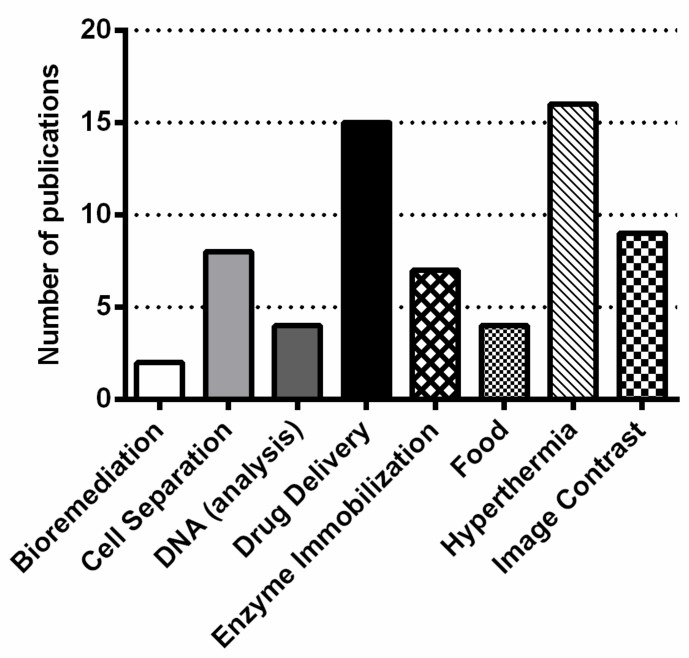
Total number of publications by application area. All scientific articles on databases [7,8] containing the keyword “magnetosome (s)” in article title were classified according to application area in Biotechnology (bioremediation, cell separation, DNA analysis, drug delivery, enzyme immobilization, food, hyperthermia, image contrast). For analysis of raw data, see Table S1. The criteria used for selection of these data are detailed in “Database generation & analysis section”.

According to our analysis, based on Web of Science [7] and Scopus [8] databases, the focuses and major efforts of studies utilizing MTB and their magnetite magnetosomes in specific applications include cell separation, hyperthermia, drug delivery and contrast enhancement of magnetic resonance imaging (Figure 3). In this mini-review, we examine the various biomedical and other applications involving the use of MTB and their magnetosomes as well as future potential uses.

## 2. Results and Discussion

### 2.1. MTB and Magnetosomes Production

#### 2.1.1. MTB and Magnetosomes

MTB are a morphologically, metabolically and phylogenetically diverse group of mostly aquatic, Gram-negative, motile prokaryotes that are ubiquitous in natural aquatic habitats [13]. The term “magnetotactic bacteria” has no true taxonomic meaning as they are distributed widely among a number of different phyla in the domain *Bacteria* [6]. The only common feature of MTB is their unusual magnetotactic behavior called magnetotaxis which is the passive alignment and motility along magnetic field lines due to the presence of magnetosomes which are intracellular, nanometer-sized magnetite (Fe_3_O_4_) and/or greigite (Fe_3_S_4_) crystals enveloped by a membrane bilayer [5]. Magnetosomes are usually arranged in one or more chains within the cell often along the cell’s long axis if the cell is not spherical in morphology [1]. The magnetosome membrane surrounding each magnetic crystal is a lipid bilayer that contains numerous proteins, which originates from invaginations of the cytoplasmic membrane of the cell [14]. The vesicle resulting from the pinching off of the membrane [15,16,17] is thought to play an important role in creating a chemical/redox environment promoting the nucleation and growth of magnetite and greigite crystals controlling their size and shape [12,18]. Most proteins shown to be involved in the biomineralization of magnetite and/or greigite are unique to MTB, many localized in the magnetosome membrane [1]. Magnetite magnetosome biomineralization is a mineralization process under strict genetic and (bio)chemical control [12,16,19]. As part of the genetic control, MTB possess specific genes called *mam* (magnetosome membrane) or *mms* (magnetic particle membrane specific) genes which encode proteins some of which are involved in magnetosome membrane formation, iron uptake, magnetic crystal nucleation and growth and assembly of the magnetosomes into chains [14,16,18,19,20,21,22,23,24]. However, some aspects and specific steps of magnetosome biomineralization are still not completely understood and may be different depending on the species of MTB [16,25,26]. In addition, other than the identification of some *mam* genes and chemical precursors, very little is known regarding greigite biomineralization in those MTB that synthesize it. In the initial step of the synthesis of magnetite magnetosomes, extracellular iron (ferric or ferrous) is taken up by the cell and passes through the outer membrane into the periplasm where magnetite crystal nucleation might occur in an invagination of the inner (cytoplasmic) membrane. Alternatively, it is possible that the iron arrives through the inner membrane and through the magnetosome membrane or is transported directly from the periplasm to the magnetosome [16]. After crystal nucleation, specific proteins regulate crystal growth, shape and size. Finally, magnetosomes are aligned within the cytoplasm of the cell in one or multiple chains. Different chemical precursors have been identified in magnetosome magnetite biomineralization, including ferrihydrite, hematite or high-spin reduced Fe complexes [1,16,25,27]. Additionally, a mechanism involving phase transformations from disordered phosphate-rich ferric hydroxide into magnetite was proposed [27]. Greigite magnetosome biomineralization precursors, including mackinawite (tetragonal FeS) and a cubic FeS, were proposed based on the study of an uncultured greigite-producing MTB [28].

Chemical methods used to produce abiotic magnetite nanoparticles include oxidative precipitation [29], thermal decomposition [30] microemulsion [31], Sol-Gel method [32] and solvothermal methods [33]. Magnetite crystals produced using these methods demonstrate that there is little, if any, control over the mineralization process, meaning that the crystals generally do not show a consistent crystal morphology or size. Advances in the characterization of the proteins involved in magnetosome formation in MTB have been applied in the development of biomimetic magnetite nanoparticles [34]. In these approaches, proteins responsible for the nucleation, pH and redox control in the biomineralization process by MTB are used in in vitro synthesis of magnetite nanoparticles to fine tune desirable crystal features.

Several of these proteins are being investigated as to their use in specific biotechnological applications. For example, one of the most abundant proteins in the magnetosome membrane of many MTB, MamC (encoded by the *mamC* gene), is strongly attached to magnetite magnetosome crystals and has been shown to be a stable anchor for a number of molecules [18,19,20,21,22,23,24]. Because only one MTB in axenic culture biomineralizes greigite (in addition to magnetite) [6] and the conditions for greigite biomineralization in this organism have not been elucidated and optimized, only magnetite-producing MTB and their magnetosomes have been used in studies investigating potential applications. Moreover, of all the MTB in axenic culture, the magnetite-producing *Magnetospirillum* species are the easiest to grow to high cell yields and are thus the most common MTB used in studies involving applications of MTB and magnetosomes [35].

**Figure 4:**
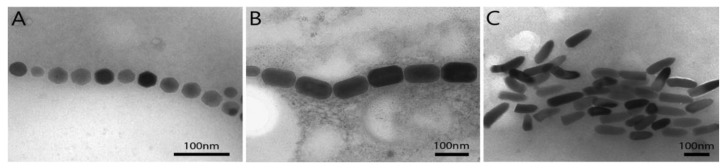
Transmission electron microscopy images of magnetosomes organized in chain(s) within magnetotactic bacteria, showing cuboctahedral (**A**), prismatic (**B**) and bullet-shaped (**C**) magnetite magnetosomes.

The magnetic mineral crystals in magnetosomes possess unique physical and magnetic features and properties which are important in their use in many applications. These crystals exhibit a consistent crystal morphology depending on the species/strain of MTB, a narrow crystal size range, relatively high chemical purity and few crystallographic defects [36,37]. The crystals habit or shape of magnetite magnetosome crystals varies between species of MTB but, in general, one species of MTB synthesizes crystals of a specific morphology [1,38]. Three general morphologies of magnetite magnetosome crystals have been found in MTB with only slight variations and include: roughly cubic (cuboctahedral) [39,40,41]; elongated prismatic (appear rectangular in projection) [4]; and bullet- or tooth-shaped (anisotropic) [42,43,44] (Figure 4). Greigite magnetosome crystals are often irregular in shape with a wrinkled surface appearance [1], although cuboctahedral and elongated prismatic greigite crystals have been observed in some uncultured MTB [45,46]. With only a few exceptions [47,48], the size of magnetosome crystals ranges from 35–120 nm [1,37], placing them in the stable single magnetic domain (SMD) size range for both magnetite and greigite. This has great significance, in that it means that magnetosome crystals are the smallest crystals of these materials that are permanently magnetic at ambient temperature without having to be placed in an external magnetic field [49,50,51,52]. Smaller particles, less than 30 nm in size, referred to as superparamagnetic, do not have a stable, remanent magnetization at ambient temperature. Cells initially produce these smaller particles, which eventually grow into mature SMD-sized crystals [46]. In crystals larger than 120 nm, domain walls tend to occur, forming multiple domains causing these crystals to be non-uniformly magnetized, thereby reducing the remanent magnetization. By biomineralizing SMD particles, MTB produce the optimum crystal size for a maximum permanent magnetic dipole moment per magnetosome [45]. The combination of these physical/chemical/magnetic characteristics, together with the enveloping magnetosome membrane, makes magnetosomes unique and worth examining for their potential in scientific, medical and other applications in biotechnology and nanotechnology. To date, only cuboctahedral magnetite nanocrystals from *Magnetospirillum* species have been studied in applications. It is conceivable however, that elongated prismatic magnetosomes from MTB, such as *Magnetovibrio blakemorei* strain MV-1, might bear greater amount of functional molecules onto its surface because the crystals’ aspect ratio (length/width) of these latter crystals is greater than that of cuboctahedral crystals [15]. Regarding composition of nanocrystals, magnetosome-inspired greigite chemically-produced nanoparticles appear to show similar magnetic properties to those of magnetite magnetosome crystals, suggesting that these iron sulfide-nanoparticles are also good candidates for biomedical applications [53].

#### 2.1.2. Magnetosome Production and Functionalization

**Figure 5:**
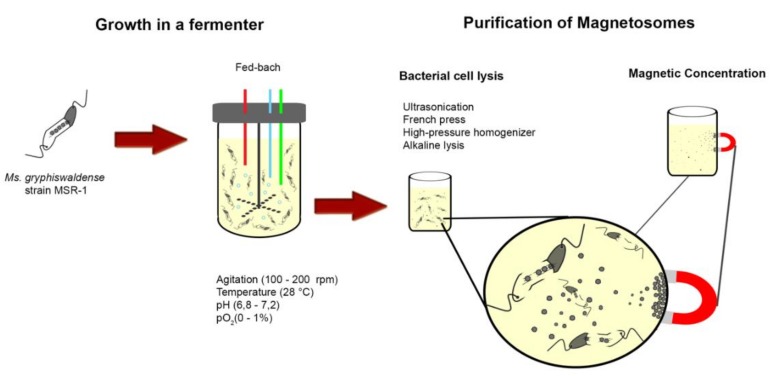
Illustrative scheme of magnetosome purification. Cells of *Magnetospirillum gryphiswaldense* strain MRS-1 are grown in a fermenter and then lysed using different methods and magnetosomes purified from lysed cells using magnetic concentration and separation. This scheme is based on numerous studies [12,56,60,61,62,63,64].

Magnetosomes have some properties superior to those found in artificially synthesized iron particles [54], including the characteristic of being dispersed easily and facile functionalization because of the magnetosome membrane [35]. However, a general problem or constraint in the production of biogenic magnetic nanoparticles is the fastidious and microaerophilic growth features of MTB [55,56]. While synthetic nanoparticles can be produced in large-scale, the yield of magnetite obtained from cultures of MTB is often low and procedures for purification of magnetosomes from cultures are considered very time-consuming [57]. Because of these problems, different strategies for cultivation of MTB in bioreactors have been proposed [56,58,59,60] and most often utilize strains of *Magnetospirillum* growing in batch, fed-batch, and semi-continuous cultures [61] (Figure 5). The pioneering work of Heyen and Schuler (2003), where they evaluated growth and magnetosomes production by *Ms. gryphiswaldense*, *Ms. magnetotacticum* and *Ms. magneticum* cultivated in a fermenter under different oxygen tensions (pO_2_), was the basis for the development of optimization strategies for culturing MTB in a bioreactor. The maximum production of magnetite in this study was achieved by growing cells of *Ms. gryphiswaldense* at a pO_2_ of 0.25 mbar (1 bar = 105 Pa) resulting in 6.3 mg of magnetite L^−1^ day^−1^ [56]. A major consideration in obtaining high cell and magnetosome yields, necessary to test them in specific applications, is the fact that relatively high O_2_ tensions (>10%) lead to accelerated cell growth but limited magnetite formation in *Magnetospirillum* species. Magnetite yields are much higher when cells are grown under very low O_2_ tensions or anaerobically [56]. Thus, strict control over O_2_ tension throughout growth is a common operational approach in large-scale cultivation of MTB [60]. Other media components, such as carbon, nitrogen and iron sources and concentrations, also have important effects on growth, biomass and magnetosome production [56,58,59]. It is important to note that, as pointed out by Xu and colleagues [57], these cultivation and magnetosome purification procedures are environmentally friendly processes with relatively good reproducibility, high yield, and low cost. 

After optimization of the protocol to yield maximum amounts of magnetosomes at low cost (which will likely take a few more years of research), the next steps to make magnetosome applications feasible are magnetosome extraction/purification and functionalization. For extraction of magnetosomes, cells of MTB must be physically or chemically lysed. Methods like ultrasonication, French press, a high-pressure homogenizer, or alkaline lysis are commonly used to cause bacterial cell lysis (Figure 5) [12,62,63,64]. More aggressive chemical or physical lysis methods would likely damage the structure of the magnetosome. For example, employing strong acids or organic solvents/detergents might cause dissolution of magnetite crystal or the removal of the phospholipid membrane, respectively. Following lysis, magnetosomes must be recovered from this cell-free extract usually employing magnetic separation techniques (applying a magnetic field to the cell lysate) thereby concentrating magnetosomes, making them easier to wash and purify (Figure 5).

The surface properties of magnetosomes makes it relatively easy to anchor significant amounts of specific molecules on to the magnetosome membrane, a great advantage for the functionalization of magnetosomes [61,65]. A number of different techniques employed the modification of proteins present in the magnetosome membrane as a starting point for functionalization. These techniques can be roughly divided into two categories: (1) genetic engineering of gene(s) that encode magnetosome surface protein(s); and (2) post-extraction chemical modification of these proteins. In the first category, some surface proteins have been shown to serve as anchors for the genetic fusion of functional peptides and proteins [66,67]. Genetic engineering can also be applied to improve properties of magnetosomes for functionalization. Well-established genetic systems for MTB are currently only available for the genetic engineering of *Magnetospirillum* species [68,69], although genetic systems are being developed for other MTB. *Magnetospirillum* species were favored in the quest for a genetic system for MTB because these species were the easiest to grow and genetically manipulate under laboratory conditions and the only available species in cell line repositories for a many years. This technology was mainly focused on *Ms. magneticum* strain AMB-1 mainly to understand the process of magnetite biomineralization although it was subsequently used in biotechnological applications, for example, to modify the surface of magnetosomes (examples are described in the next section). Recently, Mickoleit et al. (2018) produced spider silk-coated magnetosomes by expression of spider silk-inspired peptides fused to MamC in *Ms. gryphiswaldense* strain MSR-1. The authors demonstrated that the dispersability and the colloidal stability of the nanoparticles increased, and discussed that the encapsulation of magnetosomes with the polymer might improve their biocompatibility because spider silk is non-immunogenic [70]. In the second technique, the chemical functionalization of magnetosomes, cells are disrupted, the magnetosomes purified and, then functionalized by chemically coupling the molecule(s) of interest on the surface of the magnetosome membrane [63,71,72,73]. For example, crosslinking reagents like glutaraldehyde and genipin [73] can be employed to form covalent bonds between amino groups (-NH_2_) from magnetosome-surface proteins and specific functional groups present in the structure of drug molecules [63,73]. Despite the fact that most methods of magnetosome functionalization utilize amine crosslinking, the overall negative surface charge of magnetosomes, caused by the phospholipids of the magnetosome membrane, enables a strong interaction with positively charged, coating macromolecules. For example, Cheng et al. (2016) used polyethylenimine to establish a link between the anticancer recombinant plasmid phsP70-Plk1-shrNa and the magnetosome surface together with doxorubicin [74]. Poly-aminoacids, such as poly-glutamic acid, are also potential tools for conjugation functional molecules onto magnetosomes, again based on charge interactions [65]. An important limitation that can arise from surface modification of magnetosomes for drug delivery is the change in zeta potential that has been observed in different studies [73,75,76,77]. In some of these studies [75,77], the negative charge decreases from between −40 and −25 mV to between −17 and −10 mV, possibly hindering dispersibility properties that are important in pharmaceutical development. Further modifications such as the insertion of poly-l-glutamic acid (PLGA) might be useful for minimizing such changes [73].

### 2.2. Applications of MTB and Magnetosomes

As previously discussed, MTB and their magnetosomes have been evaluated as biotechnological tools in many applications. Here, we will briefly describe studies in which these magnetosomes were used in drug delivery, cell separation, food science, DNA and antigen recovery/detection, hyperthermia, MRI image contrast, enzyme immobilization and bioremediation (Table 1).

#### 2.2.1. Applications of MTB

##### MTB in Drug Delivery 

In a recent review about this particular subject, Martel (2017) discusses the use of drug-loaded MTB as “smart therapeutic agents” for an efficient delivery system that targets a specific site or organ in the body [105]. The advantage of using MTB or magnetosomes for drug delivery is that an applied magnetic field can be used to make the drug reach the specific target in the organism without affecting other, non-targeted tissues. *Magnetococcus marinus* strain MC-1 was used to transport drug-loaded nanoliposomes into hypoxic regions of colorectal tumors in mice and the results suggest an improvement in the therapeutic index of nanocarriers when associated with MTB [78]. Interestingly, it appeared that *Mc. marinus* cells were still alive and motile and exhibited both magnetotactic and aerotactic responses after being injected into the mice in the peritumoral region, reaching deeper areas of the tumor in comparison to passive agents (microspheres and dead *Mc. marinus* cells) [78]. *Mc. marinus* strain MC-1 is a marine bacterium and would not be expected to survive within a mammalian organism although this evidently was not tested or evaluated prior to this study. Another unexpected result presented by Felfoul and colleagues [78] was that *Mc. marinus* cells were clinically “safe” and did not cause negative effects when introduced in mice. This is surprising and not expected because of the general immunogenic properties of the Gram-negative bacterial cell wall [106,107]. Therefore, detailed studies of the effect of introducing MTB into living organisms should be performed, to guarantee safety. This is probably the reason why, in general, magnetosomes appear to be preferentially tested and used in any biomedical applications because these structures will not be able to multiply, cause infection nor produce a severe immunologic response, since the magnetosome membrane does not have the lipopolysaccharide of the outer membrane of the Gram-negative cell wall known to act as an endotoxin [107,108].

##### MTB in Bioremediation

Exciting approaches using live MTB and their metabolic abilities have been described for the development of new technologies in bioremediation. For example, Shimoshige et al. (2017) isolated a new strain of *Ms. magneticum* (strain RSS-1) capable of synthesizing magnetosome magnetite crystals that have a thin samarium oxide coating [79]. The co-precipitation of another mineral in the magnetosome vesicle suggests the potential use of MTB in the magnetic recovery of transition metals and synthesis of structures composed of magnetic particles and transition metals. In another study, Zhou et al. [80] used genetic engineering to improve phosphate accumulation by cells of *Ms. gryphiswaldense* in treating wastewater [80].

**Figure 6:**
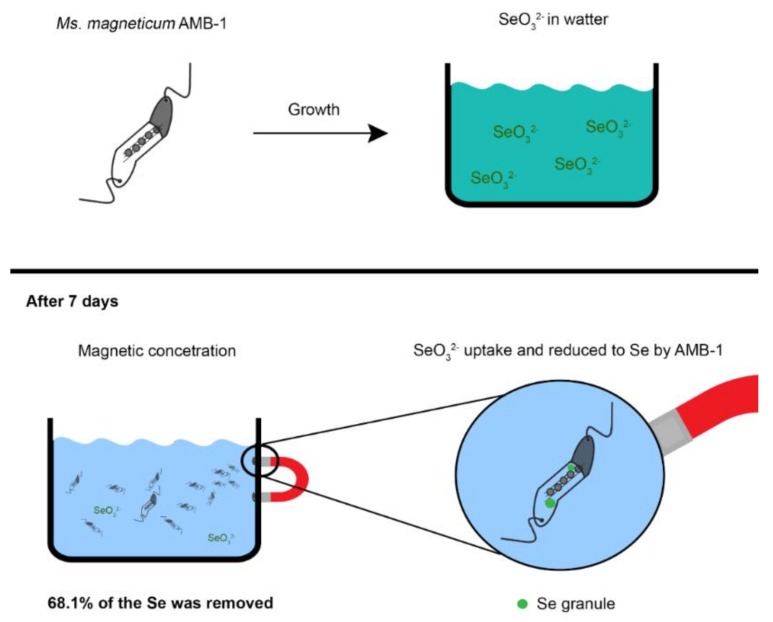
Illustrative scheme showing the magnetic recovery of Se-containing cells of *Ms. magneticum* strain AMB-1 grown in culture medium containing SeO_3_^2−^. Cells reduced SeO_3_^2−^ to Se which accumulated in cells as intracytoplasmic granules (green). After seven days of incubation followed by magnetic separation of the cells, 68.1% of the Se was removed from the medium [85].

A number of studies have demonstrated that magnetosome magnetite crystals can be doped with Cu, Mn, and Co when *Magnetospirillum* species were grown in the presence of these elements [81,82,83]. The incorporation of another transition metal in magnetosome magnetite modified the magnetic properties of the crystals and could therefore lead to the design of new biomaterials with specific, possibly tailor-made magnetic properties. Based on the absorption and immobilization of metals from culture medium and the fact that MTB can be magnetically removed from sediment and water, the use of these microorganisms in bioremediation has been proposed by several investigators [84,85,86,87]. Tanaka and colleagues [84] showed that cells of *Ms. magneticum* AMB-1 are relatively resistant to tellurium and able to concentrate and crystallize this element in structures different from magnetosomes within the cell. Unfortunately, magnetosome biomineralization was strongly affected by tellurite concentration in the growth medium, that is, cells exhibited a decreased magnetotactic response when exposed to high concentration of the element which might affect the recovery of cells using magnetic concentration techniques. The magnetic concentration and recovery of cells of *Ms. magneticum* strain AMB-1 exposed to Cd resulted in significant removal of Cd from the growth medium when tested in vitro using a genetically modified strain of the organism that had several hexahistidine residues exposed on the cell surface [109]. In the in vitro evaluation of magnetic recovery of Se using cells of *Ms. magneticum* strain AMB-1 grown in culture medium containing SeO_3_^2−^, cells were able to reduce SeO_3_^2−^ to Se, accumulating the element in intracytoplasmic granules not associated with magnetosome synthesis (Figure 6) [85]. After the magnetic recovery of cells, 68.1% of the Se was removed from the medium (Figure 6) [85]. A limiting aspect of using MTB in this type of bioremediation is that the compound or metal to be removed might also negatively affect the biomineralization of magnetosomes thus making the magnetic concentration and removal of cells difficult or impossible.

##### Use of MTB in New, Novel Technologies

Cells of MTB continue to be tested and evaluated in many novel and sometimes unusual applications. For example, Smit et al. (2018) proposed the use of MTB cells for the generation of low voltage electricity based on Faraday’s law of electromagnetic induction [88]. Blondeau et al. (2018) showed that magnetosome chain manipulation in silica-encapsulated MTB cells did not affect cell viability thereby increasing the feasibility in functional devices in the future [110]. Pierce et al. (2017) recently showed great improvements in studying the hydrodynamics of motile cells of MTB based on the control of their motility by an applied magnetic field demonstrating the potential of cell so MTB in the development of functional micro-robotic technologies [111].

#### 2.2.2. Applications of Magnetosomes

##### Magnetosomes in Drug Delivery

The use of magnetic nanoparticles as a tool for drug and gene delivery systems is one of the most studied aspects in nanotechnology and biomedicine [65]. Several research publications focus on the use of magnetosomes for this purpose and are briefly described here.

**Figure 7:**
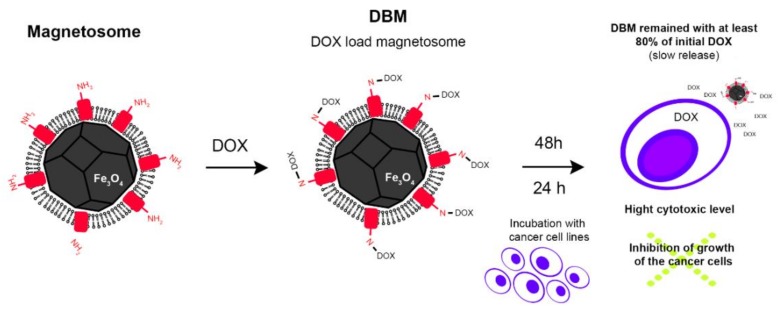
Illustrative scheme of the functionalization of magnetosome from *Ms. gryphyswaldense* strain MSR-1 with doxorubicin (DOX) binding to the amino groups of magnetosome proteins (red rectangles) inserted into the membrane of magnetosomes, forming DBM (DOX loaded magnetosomes). After treatment with DBM, cancer cells were inhibited from growing and the use of DBM resulted in a slow release of DOX. This indicates that the DBM complex is not degraded during systemic circulation and possesses potent antitumor activity [63].

A complex comprised of antitumor drug doxorubicin (DOX) and magnetosomes isolated from *Ms. gryphyswaldense* strain MSR-1 was prepared using glutaraldehyde as a crosslinking agent [112]. In this complex, 1 mg of purified magnetosomes was bound to 0.87 mg of DOX (Figure 7) [63]. The linkage between the surface of the magnetosome and DOX appeared to be very stable and the release of the drug from the complex was prolonged [63]. Antitumor activity of the magnetosome-DOX complex was examined against the HL60 and EMT-6 cell lines of human leukemia and mouse breast cancer, respectively (Figure 7) [63]. Eighty percent of the drug remained bound to the magnetosome after 48 h of incubation (Figure 7) [63]. This result indicates that this complex is not degraded during systemic circulation and most of the DOX is not significantly released before the complex reaches its target tissue. In addition, the magnetosome-DOX complex showed potent antitumor activity demonstrated by inhibition of growth of the cancer cells (Figure 7). There was no loss of antitumor activity due to any structural change caused by coupling the drug with the magnetosome [63].

In other studies involving magnetosomes in antitumor drug delivery systems, cytarabine and daunorubicin were immobilized onto magnetosomes using genipin and poly-lactide-glutamic acid crosslinking reagents [89,113]. Different preparation protocols were tested for both drugs. For cytarabine, the highest encapsulation efficiency achieved was 68.4%, which represents the maximum amount of the drug effectively bound to magnetosomes, while drug loading, defined as the ratio between the drug weight encapsulated in nanoparticles and the total weight of the nanoparticle, reached 38.9% [113]. For daunorubicin, the highest encapsulation efficiency achieved was 36.1% while drug loading peaked at 17.9% [89]. When equal amounts of free and magnetosome-attached drug were compared, the antitumor activity of the cytarabine-magnetosome complex was similar to that of the free drug. However, the magnetic complex showed a long-term drug release profile, taking 40 days of incubation for 90%-drug release from the complex [113]. This stability of the complex implies that fewer doses of the complex would be necessary for treatment [113].

Guan (2015) used a moderate ultrasonic treatment to immobilize gangliosides, which are used to treat human epidermoid carcinoma cells, onto magnetosomes [75]. Gangliosides loading efficiencies were 11.7 and 11.6 µg for ganglioside GM1 and GM3, respectively [75]. Immobilization of GM1 onto the surface of magnetosomes significantly increased ganglioside uptake by YTS-1 carcinoma cells. Immobilization of GM3 increased the inhibition of the activation of epidermic growth factor receptors (EGFR) in human epidermoid carcinoma cells. Both effects were greater when a magnetic field was applied in the experiments [75], indicating that the presence of the magnetic field enhanced penetration of functionalized magnetosomes into the cells.

Apart from directly delivering drugs, magnetosomes have been employed in gene delivery to enhance drug-mediated cancer treatment. Wang et al (2018) recently described a strategy for the treatment of human hepatocellular carcinoma (HepG2 cells) in which magnetosomes were functionalized with the plasmid pVAX1-VA that encodes for two anti-tumor molecules, cecropin B and apoptin. This approach resulted in the efficient delivery of pVAX1-VA into HepG2 cells and increased tumor inhibition in vivo by enhancing membrane permeability and upregulation of caspases [90].

##### Magnetosomes in Cell Separation

In 1987, Matsunaga and colleagues demonstrated that magnetosomes could be introduced into red blood cells (RBC) by cell fusion using polyethylene glycol [114]. MTB have also been used to separate leucocytes based on their phagocytosis profile [115]. After phagocytosis of MTB and exposing the cells to a magnetic field, up to 95% of the leucocytes were efficiently concentrated and separated from samples containing cells that had not ingested MTB [115]. Each leucocyte contained approximately 20–40 MTB cells and displayed high viability, chemotactic and phagocytic abilities [115].

Magnetic cell separation using magnetosomes and specific antibodies has also been achieved. For example, Yoshino (2008) designed reconstructed magnetosomes expressing antibody-linking protein A on their surface for cell separation [91]. The expression of the functional protein was achieved by the introduction of a plasmid containing the gene encoding the magnetosome membrane protein MamC fused to the gene for the ZZ domain of protein A in cells of *Ms. magneticum* strain AMB-1 [91]. The modified magnetosomes were bound to anti-murine G immunoglobulins (Ig) and used for sorting peripheral blood cells that were pretreated with anti-CD14, CD19 and CD20 murine monoclonal antibodies [91]. Monocytes (CD14+) and B-lymphocytes (CD19+ and CD20+) were separated and recovered with efficiencies of 95.7%, 97.2% and 98.8%, respectively [91].

Takahashi et al. (2010) developed a multifunctional magnetic nanoparticle with the introduction of a polypeptide bridge consisting of repeated units of asparagine and serine residues (NS polypeptide) and the A domain of Ig-binding protein G [92]. The polypeptide was bound to the surface of magnetosomes by genetic fusion of the polypeptide-encoding gene to the *mamC* gene, as well as that that encodes protein G [92]. The NS polypeptide worked as a barrier to avoid interaction between magnetosomes which might lead to their aggregation [92]. Moreover, this polypeptide barrier prevented non-specific interactions between magnetosomes and immune system cells, for example, macrophages and T-lymphocytes [92]. Ultimately, magnetosomes were bound to antibodies to magnetically separate cells from peripheral blood [114]. Non-target cells were separated to a much lesser extent than when magnetosome-protein G complexes without the polypeptide bridge were used [92]. The new complex displayed superior specificity and dispersibility characteristics [92].

Ig-magnetosome complexes have also been prepared by mixing magnetosomes extracted from *Magnetospirillum* strain SO-1 with an immunocomplex rather than expressing fusion vectors into cells of this MTB [93]. Two different fusion proteins from *Staphylococcus aureus* (Mbb) and *Bacillus subtilis* (Mistbb) were prepared after expression in *Escherichia coli*. Mbb consisted of the double-Ig-binding domain (BB-domain) of the staphylococcal protein A in the MamC protein from *Mc. marinus* strain MC-1 [93]. Mistbb consisted of the BB-domain, an Ig-binding domain, but the transmembrane domain was Mistic proteins (MistBB) from *B. subtilis*. Both proteins displayed similar Ig-G binding activity [93]. Mbb and MistBB were then inserted into the magnetosome membrane through vortexing and ultrasonication under different experimental conditions. Ultrasonication resulted in a higher level of integration of the fusion proteins into the magnetosome membrane [93]. Thus, the insertion of a protein with an Ig-binding domain on the surface of magnetosomes might simplify the functionalization process [93].

##### Magnetosomes in Food Safety

Functionalized magnetosomes have been used to efficiently detect pathogens in food. For instance, Xu and colleges constructed a capture system with the recombinant magnetosome isolated from cells of *Ms. gryphiswaldense* strain MSR-1 in which the protein A gene was fused to the *mamC* gene [94]. The complex was bound to a specific antibody to capture *Vibrio parahaemolyticus*, a pathogen that causes many gastrointestinal, foodborne illnesses, from foods [94]. One milligram of this complex was able to capture 1.74 × 10^7^ cells of the pathogen [94].

In a similar application, Li et al. (2010) created a magnetosome-polyclonal antibody complex to capture cells of *Salmonella* species from food [76]. However, instead of using genetic engineering techniques, the cross-linking reagent bis(sulfosuccinimidyl) suberate (BS3) was employed for the attachment of specific antibodies onto the surface of magnetosomes. In this study, 178 μg of antibody was immobilized onto 1 mg of magnetosomes [76]. When used for the detection and separation of *Salmonella dublin* from a test suspension, the capture efficiency was as high as 87%, as measured by fluorescence quantitative-PCR [76]. Pathogen detection using this complex was also determined in food samples. *Salmonella* was detected in artificially contaminated food samples (e.g., milk, eggs and pork) when the pathogen was present in concentrations higher than 60 CFU/mL [76]. This magnetosome-antibody complex also showed high specificity, as it was unable to capture *Vibrio* cells from mixed suspensions of *Salmonella* and *Vibrio* [76].

A magnetosome-anti-enterotoxin-Ig complex, was developed and used as a biosensor for the detection of *Staphylococcus aureus* enterotoxin in contaminated milk [77]. This biosensor was constructed by immobilizing a film of immunoglobulin-functionalized magnetosomes on a gold electrode and was used to evaluate concentrations of *S. aureus* enterotoxin in artificially contaminated milk samples [77]. This biosensor showed a reasonably linear response and a wide range of concentration measuring ability; 88–118% of the enterotoxin was detected with a correlation coefficient of 0.9957 [77]. The biosensor with magnetosomes had lower detection limit (0.017 ng/mL) compared to another without magnetosomes (0.033 ng/mL) [77].

##### Magnetosomes in DNA and Antigen Recovery/Detection Assays

Magnetosomes were successfully used as a component in protein detection assays [71]. Biotin groups coupled to the magnetosome membrane on magnetosomes were used for the attachment of the protein streptavidin [71]. These semisynthetic composite particles with a specific biotin-binding capacity could be used to link several functional biomolecules, such as biotinylated DNA oligonucleotides or biotinylated antibodies [71]. This technology is may be important in improving immunological diagnostics and proteome research [58]. Following this technology, a modification in an automatable, highly sensitive immuno-PCR (M-IPCR) was created using antibody-functionalized magnetosomes in a surface-independent immunoassay [72]. In this technique, antibody-functionalized magnetosomes were used for the immobilization of HBsAg (hepatitis B antigen) in human serum and enhancement of the generated signal by the detection complex through magnetic concentration [72]. The detection of HBsAg using the M-IPCR was about 100-fold more sensitive than magneto-ELISA (Enzyme Linked Immunosorbent Assay), which uses synthetic nanoparticles to enhance antigen detection in ELISA and was performed in parallel to M-IPCR for comparison purposes [72].

##### Magnetosomes as Magnetic Resonance Imaging (MRI) Contrast Agents

Herborn and colleagues (2003) characterized magnetosomes as superparamagnetic contrast agents for magnetic resonance imaging (MRI) in cell cultures and animal models [116]. Following this, the potential use of magnetosomes as MRI image contrast agents was demonstrated in several cell model including pancreatic cells [95], brain cells [96,97], mammalian cells [98], cells of xenograft tumors [97,99] and breast cancer cells [99]. In almost all cases magnetosomes proved to highly promising tools for the detection of and treatment of tumors using hyperthermia. The main advantages of using magnetosomes were that a relatively low dosage of magnetosomes could be used [117] and the high affinity of magnetosomes to target cells because of specific proteins bound to the surface of magnetosomes [99].

Recently, Kraupner and colleagues [118] compared the use of magnetosomes as a magnetic tracer material in a new diagnostic imaging technology called magnetic particle imaging (MPI), to the use of the gold standard commercial tracer Resovist^®^. Results showed a significant increase in particle detection and, consequently, improvement in the resolution of the technique when magnetosomes were applied [118].

##### Magnetosomes in Hyperthermia

About 60 years ago, Gilchrist and colleagues [119] described the use of 20–100 nm diameter hematite (Fe_2_O_3_) magnetic particles and application of an altering magnetic field at 1.2 MHz to induce heat in lymph nodes and cause lymphatic metastases death [119]. The use of hyperthermia in the treatment of cancers is appealing because it does not have the toxic side effects and thus is less restrictive than chemotherapy and radiotherapy, and it could even be used in combination with these therapies thereby increasing treatment efficiency [120]. In the hyperthermia procedure, tumor cells are destroyed and the tumor reduced in size or eliminated completely by increasing the temperature within the tumor typically within the range of 37–45 °C [74,100,101,121,122,123,124,125]. Superparamagnetic iron oxide nanoparticles (SION) are artificially synthesized nanoparticles used for hyperthermia and are often limited in effectiveness by the inability to place them specifically and solely within the targeted tumor tissue. As a result, SION carry a high risk of side effects when introduced in an organism [101,120]. As discussed elsewhere in this review, due to the lipid membrane surrounding magnetosomes, it is possible to bind proteins to it that recognize specific cells and tissues in the organism. Thus, any treatment that uses magnetosomes as delivery systems or in hyperthermia could be made to be selective to damaged or cancerous tissues [35,122,123,124,125,126]. Magnetosomes are therefore regarded as an excellent alternative to SION in hyperthermia therapies [127]. Moreover, the use of an alternating magnetic field also has the potential to control the release of drugs from functionalized magnetosomes [128].

The use of magnetosomes in the hyperthermia treatment of tumors has been demonstrated [101]. Suspensions of individual, separated magnetosomes and chains of magnetosomes were introduced to a tumor consisting of cells from the MDA-MB-231 breast cancer cell line induced under the skin of mice. An applied magnetic field of 20 mT with frequency of 198 kHz for 20 min resulted in the temperature of tumor to reach 43 °C. Magnetosome chains resulted in a higher efficiency in the killing of tumor cells than individual, separated magnetosomes. The use of chains of magnetosomes led to a complete elimination of the tumor within 30 days while the use of individual magnetosomes showed significant antitumor activity. This difference was attributed to a better distribution of the internalized magnetosome chains within the cells than individual magnetosomes. The efficiency of artificially-synthesized superparamagnetic nanoparticles was also lower than both individual magnetosomes and magnetosome chains. Using a similar approach, Mannucci and colleagues [102] injected suspensions of magnetosomes into a xenografted model of a glioblastoma comprised of U87MG cells in mice and then exposed the mice to an alternating magnetic field (29 mT, 110 kHz) six times (20 min each session) in a period of two weeks [102]. Histological analyses revealed the accumulation of magnetosomes into the parenchymal tissue of tumors. Following this treatment, necrotic tissue was clearly evident in areas surrounding clusters of magnetosomes and tumor growth was significantly inhibited [102].

**Figure 8:**
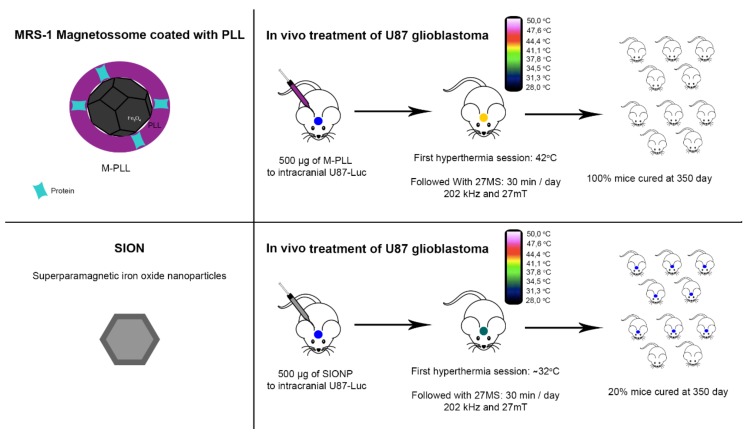
Illustrative scheme of cancer hyperthermia therapy research protocols using magnetosome magnetite crystals [104]. Magnetosomes coated with poly-l-lysine (PLL) without membrane (M-PLL) presented an antitumor effect in intracranial U87-Luc tumors in mice. This effect was observed after treatment of mice with 500 μg of M-PLL followed by 28 magnetic sessions (MS) 30 min/day with 202 kHz and 27 mT. In this case, hyperthermia induced a temperature increase, reaching 42 °C. After 350 days of the first exposure, tumors were eliminated in 100% of the treated mice. In contrast, the same treatment using superparamagnetic iron oxide nanoparticles (SION) resulted in a less effective increase of temperature and only 20% mice had tumors eliminated after 350 days [100].

Recently it was shown that magnetosomes coated with poly-l-lysine (PLL) are more stable, non-pyrogenic and have a higher potential for generating heat leading to a significantly improved antitumor effect in intracranial U87-Luc tumors in mice (Figure 8) [100]. Hyperthermia treatment with magnetosomes in this case, induced a temperature increase of 42 °C in the tumor during the 28 magnetic exposures (Figure 8) [100]. After 68 days of the first exposure, tumors were eliminated in 100% of the treated mice. In contrast, only 20% of tumors were eliminated in animals treated with SION [100]. This antitumor cell activity was confirmed by histological analyses clearly demonstrating that treatment with magnetosomes is more efficient compared to hyperthermia treatment with SION [100]. On the other hand, photothermal treatment showed superior inhibition of PC3 cancer cells (human prostate cancer cell line) than magnetic hyperthermia with magnetosomes [129]. However, in vivo assays showed strong tumor inhibition when genetically-engineered magnetosomes, modified by the fusion of an arginine-glycine-aspartic acid peptide coding gene to *mamC*, were administrated systemically and submitted to laser excitation [129].

##### Magnetosomes in Enzyme Immobilization

Magnetic nanoparticles, including magnetosomes, have recently been a popular choice as a support material for enzyme immobilization, mainly due to the simplicity of the recovery process by magnetic separation [130]. The protein display system of magnetosomes can be used for the expression of catalytic units, making them ideal candidates for the support of immobilized enzymes, as previously discussed. Ginet and colleagues [103] reported the expression of an organophosphohydrolase from *Flavobacterium* sp. fused to MamC for the degradation of paraoxon, a toxic but commonly used pesticide. This protein complex showed had a paraoxon degradation activity rate similar to that of purified organophosphohydrolase. Additionally, the authors examined the stability of the magnetosome enzyme-complex and demonstrated that the complex retained between 90% and 100% of its activity after three cycles of usage [103]. This work clearly demonstrates the potential use of magnetosomes in bioremediation.

Honda et al. (2015) explored the potential use of magnetosome-enzyme complexes in the production of biofuels [104]. In this work, a multi-enzyme complex was constructed on the surface of magnetosomes. The complex comprised adherence peptides genetically-fused to MamC through a peptide bridge, which, in turn, were used to attach the enzymes endoglucanase and beta-oxidase. Cellulose degradation activity of this complex was significantly higher than that of the two non-immobilized enzymes assayed individually. Moreover, 70% of the cellulose degradation activity of the multi-enzymatic complex was retained after five cycles of utilization.

## 3. Conclusions and Future Perspectives

Several years following the discovery and description of MTB and magnetosomes in 1975 [4], many researchers became interested in their potential use(s) in biotechnological and nanotechnological applications because of the magnetic, physical and optical properties of MTB and magnetosomes. Subsequent studies eventually leading to more efficient and less expensive methods of magnetosome purification have now led to the testing of MTB and magnetosomes in specific biotechnological and nanotechnological applications, some of which are described in this review. Because chemically-synthesized magnetic nanoparticles had already been used in a number of applications, many of the recent studies include a comparison of these particles with magnetosomes. Results obtained in many of these studies demonstrate several advantages of using magnetosomes and magnetosome magnetite crystals in a number of biotechnological and nanotechnological applications. These advantages include: (i) uniform particle shape and size; (ii) high chemical purity; (iii) unique magnetic properties (they are single magnetic domains); (iv) an apparent low toxicity; and (v) the possibility of bioengineering and functionalization due to the magnetosome membrane. Progresses in the understanding of magnetosome magnetite biomineralization and the development of different methods for their functionalization has greatly increased the potential for magnetosomes in many additional applications.

Advances in the molecular biology and genetics involved in magnetosome magnetite biomineralization resulted in numerous protocols and DNA recombinant technologies to functionalize magnetosomes, thereby improving their use in biomedicine and bioremediation. In addition, many recent advances in culturing MTB, particularly after 2013, appear to have had a significant impact on the use of magnetosomes in biotechnological applications. Likewise, the ability to transfer the genes for magnetosome magnetite biomineralization to other microorganisms will very likely lead to increased, less expensive methods of magnetosome production and purification, thereby promoting additional potentials and possibilities in the use of magnetosomes in biotechnological and nanotechnological applications [131]. Finally, improvements in the chemical modification of magnetosomes will likely also result in the development novel magnetosome-based nanoparticles useful in medicine, as sensors and in imaging, for example, the fluorescent magnetic nanoparticles designed by [132].

**Figure 9:**
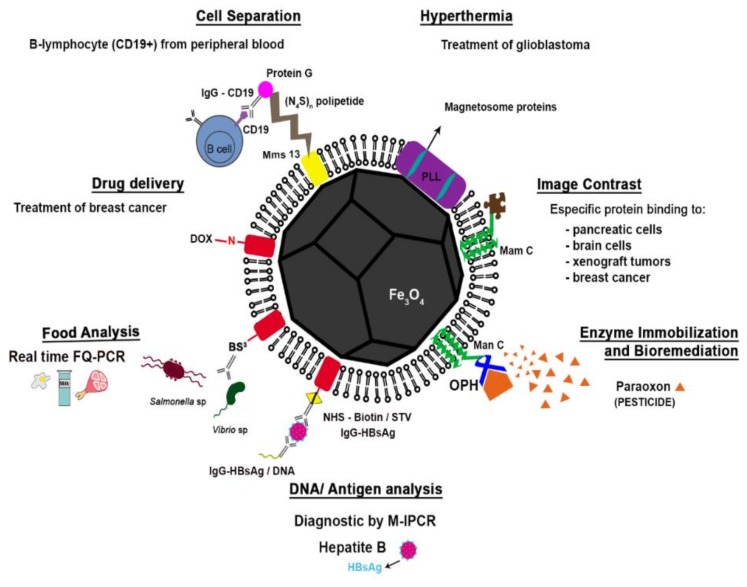
Schematic representation of a functionalized magnetosome according to each application described in the Biotechnology section of this review (cell separation; hyperthermia, drug delivery, image contrast, food analysis, enzyme immobilization and bioremediation and DNA and antigen recovery/detection). Drug delivery: the association between the surface proteins of the magnetosome and doxorubicin (DOX), an anti-breast cancer drug [63]. Cell separation: the modified magnetosomes were bound to anti-murinic G Ig anti-CD19 and used for separating B-lymphocytes from peripheral blood cells [90]. Food sciences: a capture system with the magnetosome proteins fused using a cross-linking reagent bis(sulfosuccinimidyl) suberate (BS3) for attachment of antibodies to *Salmonella* and *Vibrio* species from food samples (e.g., milk, egg and pork) [76]. DNA/Antigen analysis: antibody-functionalized magnetosomes were used for immobilization of HBsAg (hepatitis B antigen) in human serum and enhancement of sensitivity of immunoassay [72]. Image contrast: magnetosomes with specific proteins bound to the surface with high affinity to target cells were used as superparamagnetic contrast agents for magnetic resonance imaging [117]. Hyperthermia: magnetosomes coated with poly-l-lysine (PLL) were used in hyperthermia [100]. Enzyme immobilization and bioremediation: magnetosome expressing MamC fused with organophosphohydrolase (OPD) from of *Flavobacterium* sp., were used for the degradation of paraoxon [103].

Given that we have now reached the point where MTB and magnetosomes are now being tested in numerous biomedical applications, as shown and described in this mini-review (Figure 9), it is now necessary to fully understand the effects of the introducing MTB and magnetosomes into host cells and organisms. Some questions that should be addressed regarding the impact of magnetosomes on health are: (1) how toxic are introduced magnetosomes to different types of cell lineages?; (2) how are magnetosomes processed by cells after internalization?; (3) how do specific types of cells (e.g., kidney, liver) in an organism process magnetosomes if administered in different ways?; and (4) can magnetosomes across the blood-brain barrier and can they cause damage to the brain? Answering these questions will involve short- and long-term studies but, once addressed, surely the use of MTB and magnetosomes in biomedical procedures will increase was as procedures in limiting any toxic effects that might be found. Other areas that need to be investigated are the roles of MTB in heavy metal uptake and metal processing in magnetosome magnetite biomineralization to fully evaluate the potential and impact of MTB in bioremediation. It is now very clear that MTB have not only been shown to have a significant impact in environmental biogeochemistry but also in applied biotechnology, nanotechnology and medicine.

## Figures and Tables

**Table 1:** Summary of biotechnological applications of whole magnetotactic bacteria (MTB) cells and magnetosomes comparing advantages and limitations of each approach.

**Applications of whole MTB**						
**Field**	**Application**			**Ref.**	**Advantages**	**Disadvantages**
Drug delivery	Drug-loaded nanoliposomes attached to *Mc*. *marinus* cells for targeted tumor treatment			[78]	Dispenses cell lysis; Uses cell’s own magnetotaxis	Potentially immunogenic due to outer LPS
Bioremediation	Wastewater treatment; Removal of heavy metals (Cd, Te, Se)			[79,80,81,82,83,84,85,86,87]	Magnetic crystal doping possible; Recovery of removed minerals	Poor growth of MTB in contaminated media; Biomineralization may be affected
Energy generation	Electricity generation by cells and magnetosomes of *Ms. magneticum* AMB-1 by means of electromagnetic induction			[88]	Green energy technology	Only millivolts generated; Expensive
**Applications of magnetosomes**						
**Field**	**Application**	**Ref.**	**Functionalization Method**		**Advantages**	**Disadvantages**
Drug delivery	Delivery of antitumor drugs: doxorubicin, cytarabine, daunorubicin; delivery of gangliosides; Antitumor gene delivery	[63,74,75,89,90]	Chemical crosslinking with glutaraldehyde and genipin/PLGA; Surface adsorption of plasmids		Targeted drug delivery; Reduction of drug toxicity; Tissue specificity; Easy functionalization	Possible activity alteration; Unclear biological fate; Endotoxin test needed
Cell separation	Sorting of blood cells;	[91,92,93]	Binding protein expression by vector cloning; Insertion of modified binding protein into membrane		Reutilization of capture complex; High specificity separation	Difficult steps of cloning and expression; Alteration of cell viability after capture
Food safety	Capture of Salmonella and Vibrio cells; Enterotoxin detection	[76,77,94]	Crosslinking of antibodies		Reutilization of capture complex; High sensitivity	Antibody specificity
MRI contrast agent	Diagnostic detection of tumors	[95,96,97,98,99]	No functionalization; Chemical coupling of targeting peptide		May also be used as therapeutic tool (by hyperthermia, drug delivery); High affinity to target cells; High detection sensitivity	Unclear biological fate; Endotoxin test needed
DNA/Antigen Recovery/Detection	Capture of oligonucleotides and antibodies; Hepatitis B antigen detection	[71,72]	Biotinylation by chemical crosslinking with NHS		High sensitivity and recovery efficiency	Complex technology
Hyperthermia	Treatment of tumors	[100,101,102]	No functionalization, generally		Less significant side-effects than chemotherapy and radiotherapy; Tissue specificity; May also be used as diagnostic tool	Unclear biological fate; Endotoxin test needed
Enzyme immobilization	Bioremediation of organophosphate pesticides; Cellulose degradation	[103,104]	Enzyme expression by vector cloning		Reutilization of nanobiocatalyst; Immobilization of multiple catalysts	Difficult steps of cloning and expression; Possible loss of activity due to immobilization

## Supplementary Materials

The following are available online. Database generation & analysis section: reference databases were generated using the Web of Science and Scopus. The first searches (Figure 1) were performed using “magnetotactic bacteria or magnetosome(s)” in article titles, the second (Figure 2) was performed using combining “magnetotactic bacteria or magnetosome(s)” in article “Title” with words (image contrast, hyperthermia, food, drug delivery, DNA analysis, cell separation and bioremediation) in the “Title”, “Abstract” and “Key words” tools. Timespan was limited from 1975 (January 1st) to 2018 (February 25th). Therefore, to avoid literature confusion and errors, for each list of results obtained with the parameters described above, the “Create Citation Report” tool, available at the both of database web site, was used and allowed to create and export a table using the EndNote software (version X6) (Supplementary Tables S1 and S2). Duplicated references were eliminated. Results were plotted using GraphPad Prism software (version 6.0) and Microsoft Office ExcelTM (version 2007).
